# Photophysical Property and Photocatalytic Activity of New Gd_2_InSbO_7_ and Gd_2_FeSbO_7_ Compounds under Visible Light Irradiation

**DOI:** 10.3390/ijms14010999

**Published:** 2013-01-07

**Authors:** Jingfei Luan, Yong Xu

**Affiliations:** State Key Laboratory of Pollution Control and Resource Reuse, School of the Environment, Nanjing University, Nanjing 210093, Jiangsu, China; E-Mail: xuscut2006@163.com

**Keywords:** inorganic compounds, crystal growth, X-ray diffraction, catalytic properties, optical properties

## Abstract

Gd_2_InSbO_7_ and Gd_2_FeSbO_7_ were synthesized first, and their structural and photocatalytic properties were studied. The lattice parameters and the band gaps for Gd_2_InSbO_7_ and Gd_2_FeSbO_7_ were 10.449546 Å, 10.276026 Å, 2.897 eV and 2.151 eV. The photocatalytic degradation of rhodamine B was performed with Gd_2_InSbO_7_ and Gd_2_FeSbO_7_ under visible light irradiation. Gd_2_InSbO_7_ and Gd_2_FeSbO_7_ had higher catalytic activity compared with Bi_2_InTaO_7_. Gd_2_FeSbO_7_ exhibited higher catalytic activity than Gd_2_InSbO_7_. The photocatalytic degradation of rhodamine B followed with the first-order reaction kinetics, and the first-order rate constant *k* was 0.01606, 0.02220 or 0.00329 min^−1^ with Gd_2_InSbO_7_, Gd_2_FeSbO_7_ or Bi_2_InTaO_7_ as photocatalyst. Complete removal of rhodamine B was observed after visible light irradiation for 225 min or 260 min with Gd_2_FeSbO_7_ or Gd_2_InSbO_7_ as photocatalyst. The evolution of CO_2_ was realized, and it indicated continuous mineralization of rhodamine B during the photocatalytic process. The possible photocatalytic degradation pathway of rhodamine B was proposed.

## 1. Introduction

Nowadays, with the development of industry, wastewater is yielded in bulk. Particularly, some industries, such as textile and dyeing manufacturing, produce a large amount of dyestuff wastewater [1]. These dyestuffs are usually synthetic aromatic compounds, which are seriously harmful to the aquatic biota. Moreover, the coloration of the wastewater in streams or lakes would absolutely influence the solar illumination, which would affect the growth of creatures and hydrophytes. Among all kinds of dyestuff, rhodamine B (RhB) is one of the most important representatives of xanthene dyes. RhB is toxic and resistant to biodegradation and direct photolysis, thus RhB would have a considerable deleterious effect upon the environmental matrix. RhB undergoes natural reductive anaerobic degradation; as a result, potentially carcinogenic aromatic amines [2,3] are yielded. Moreover, RhB is widely used as a photosensitizer, a quantum counter and an active medium in dye lasers, *etc*. [4,5]. Thus, we use RhB as a probe contaminant to evaluate the activity of new photocatalysts, both under ultraviolet light and visible light irradiation.

In terms of the enormous harm of RhB dye wastewater, it was thoroughly urgent to remove RhB dye from effluents before they were emitted to natural water bodies. At present, several treatment measurements were adopted for RhB dye removal. For example, the technology of adsorption using absorbents [3,6–8], such as activated carbon, has been found to be an efficient technology for decolorization of wastewater. Although it was quite effective for activated carbon to adsorb RhB dye in wastewater, activated carbon was restricted from use because of its high cost and difficulty in being regenerated. Therefore, a novel processing method, photocatalysis, has been developed for removing RhB dye wastewater. Photocatalysis is an advanced oxidation processes and has aroused more and more attention by investigators [9–23], and much scientific research on the photocatalytic degradation of aqueous organic contaminants has been reported [10–31]. Some investigations [2,32–38] about the photodegradation of RhB have been reported under ultraviolet light or visible light irradiation, showing that photocatalysis was an effective degradation method for degrading dye to a large extent.

With the development of the investigation of the photocatalysis process, investigators also paid much attention to researching and developing novel photocatalysts [39–43]. Presently, TiO_2_ was the most common photocatalyst; however, TiO_2_ could not be used in the visible light region and could only degrade RhB under ultraviolet light irradiation. Moreover, ultraviolet light only occupied 4% of sunlight, which was a restrained factor for photocatalysis technology with TiO_2_ as the catalyst. Therefore, some efficient catalysts, which could generate electron-hole pairs under visible light irradiation, should be developed, because visible light occupies 43% of sunlight. Fortunately, A_2_B_2_O_7_ compounds were often considered to have photocatalytic properties under visible light irradiation. In our previous work [44], we have found that Bi_2_InTaO_7_ crystallized with the pyrochlore-type structure and acted as a photocatalyst under visible light irradiation and seemed to have a potential for improvement of the photocatalytic activity upon modification of its structure. Based on the above analysis, we could assume that substitution of Ta^5+^ by Sb^5+^, substitution of Bi^3+^ by Gd^3+^ and substitution of In^3+^ by Fe^3+^ in Bi_2_InTaO_7_ might increase carrier concentration; as a result, the new photocatalysts Gd_2_InSbO_7_ and Gd_2_FeSbO_7_ might have advanced photocatalytic properties. Gd_2_InSbO_7_ and Gd_2_FeSbO_7_ were never synthesized before and never used in the photocatalysis progress. The molecular composition of Gd_2_InSbO_7_ and Gd_2_FeSbO_7_ was very similar with other A_2_B_2_O_7_ compounds. Thus, the resemblance suggested that Gd_2_InSbO_7_ and Gd_2_FeSbO_7_ might possess photocatalytic properties under visible light irradiation, which was similar with the other members in the A_2_B_2_O_7_ family. This paper reported the preparation process and property characterization of Gd_2_InSbO_7_ and Gd_2_FeSbO_7_. Both Gd_2_InSbO_7_ and Gd_2_FeSbO_7_ were semiconductor compounds that were synthesized for the first time. In this contribution, we discussed the structural and photocatalytic properties of Gd_2_InSbO_7_ and Gd_2_FeSbO_7_ by degrading RhB under visible light irradiation and compared the photocatalytic activity among Gd_2_InSbO_7_, Gd_2_FeSbO_7_ and Bi_2_InTaO_7_ in order to elucidate the structure-photocatalytic activity relationship in these newly synthesized compounds.

## 2. Results and Discussion

### 2.1. Characterization

**Figure 1:**
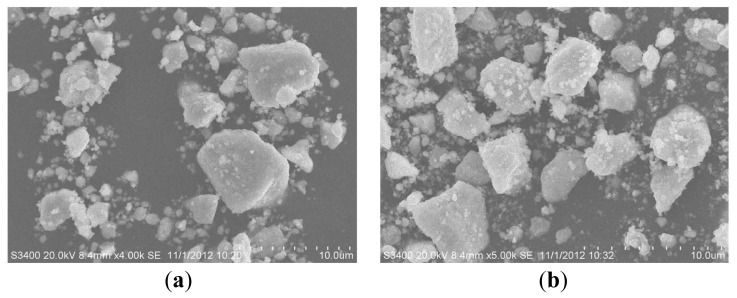

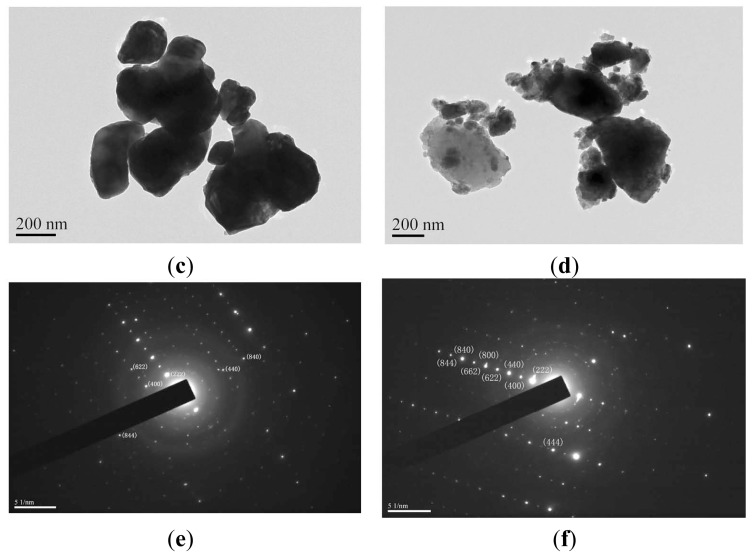
(**a**) Scanning electron microscope (SEM) image of Gd_2_InSbO_7_ with low magnification; (**b**) SEM image of Gd_2_FeSbO_7_ with low magnification; (**c**) Transmission electron microscopy (TEM) image of Gd_2_InSbO_7_ with high magnification; (**d**) TEM image of Gd_2_FeSbO_7_ with high magnification; (**e**) The selected area electron diffraction (SAED) pattern of Gd_2_InSbO_7_; (**f**) The SAED pattern of Gd_2_FeSbO_7_.

Figure 1a,b show the SEM images of Gd_2_InSbO_7_ and Gd_2_FeSbO_7_ with low magnification. Figure 1c,d show the TEM images of Gd_2_InSbO_7_ and Gd_2_FeSbO_7_ with high magnification. The results showed that the particle diameter of Gd_2_FeSbO_7_ was 240 nm and the particle diameter of Gd_2_InSbO_7_ was 310 nm. The nanosized particles of Gd_2_FeSbO_7_ and Gd_2_InSbO_7_ were obtained, and these particles were in inhomogenous global shapes. Figure 1 revealed that the average particle size of Gd_2_FeSbO_7_ was smaller than that of Gd_2_InSbO_7_. There were two reasons to obtain nanocrystals in this experiment, while synthesizing Gd_2_InSbO_7_ and Gd_2_FeSbO_7_ powders by the solid-state method. Firstly, the powders, which were obtained after a sintering process by an electric furnace, would be ground by mortar three times and then would be ground in a ball mill three times. The total time for grinding the above photocatalyst powders was approximately 15 h. Secondly, the sizes of the photocatalyst particles were different. We utilized a centrifuge to realize separation of the photocatalyst particles, which had different sizes by adjusting the rotation speed of the centrifuge, and the small particles were obtained. SEM-EDS spectrum taken from the prepared Gd_2_FeSbO_7_ indicated the existence of gadolinium, iron, antimony and oxygen. As to the prepared Gd_2_InSbO_7_, gadolinium, indium, antimony and oxygen were detected and other elements were not identified.

**Figure 2:**
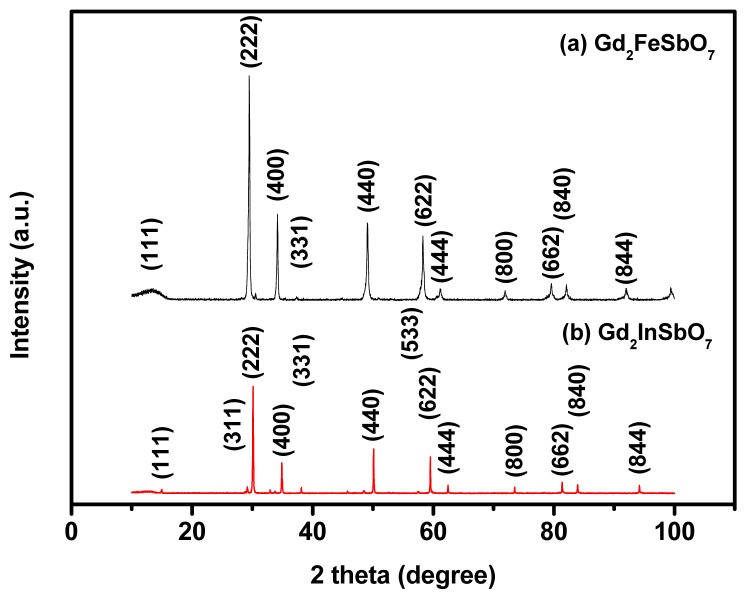
X-ray powder diffraction patterns of (**a**) Gd_2_FeSbO_7_ and (**b**) Gd_2_InSbO_7_.

**Figure 3:**
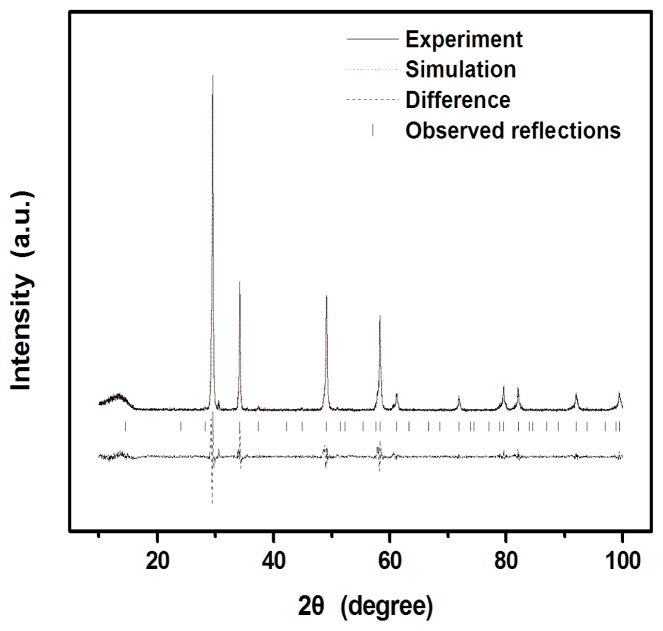
X-ray powder diffraction patterns and Rietveld refinements of Gd_2_InSbO_7_ prepared by a solid-state reaction method at 1320 °C. A difference (observed-calculated) profile is shown beneath. The tic marks represent reflection positions.

X-ray powder diffraction patterns of Gd_2_FeSbO_7_ and Gd_2_InSbO_7_ are shown in Figure 2. Figure 3 displays the powder X-ray diffraction patterns of Gd_2_InSbO_7_ together with full-profile structure refinements of the collected data as obtained by the RIETAN™ [45] program, which is based on Rietveld analysis. The results of the final refinement for Gd_2_FeSbO_7_ and Gd_2_InSbO_7_ manifested an excellent consistency between the observed and calculated intensities for the pyrochlore-type structure, a cubic crystal system and a space group *Fd3m* (O atoms were included in the model). The lattice parameters of Gd_2_InSbO_7_ and Gd_2_FeSbO_7_ were 10.449546 Å and 10.276026 Å, respectively. All the diffraction peaks for Gd_2_FeSbO_7_ and Gd_2_InSbO_7_ could be successfully indexed based on the lattice constant and above space group. The atomic coordinates and structural parameters of Gd_2_FeSbO_7_ and Gd_2_InSbO_7_ are listed in Tables 1 and 2, respectively. In addition, Our XRD results showed that Gd_2_FeSbO_7_ and Gd_2_InSbO_7_ crystallized with the same structure, and 2 theta angles of each reflection of Gd_2_InSbO_7_ changed with the substitution of In^3+^ by Fe^3+^. The lattice parameter decreased from *α* = 10.449546 Å for Gd_2_InSbO_7_ to *α* = 10.276026 Å for Gd_2_FeSbO_7_, indicating a decrease in the lattice parameter of the photocatalyst with the decrease of M ionic radii, Fe^3+^ (0.78 Å) < In^3+^ (0.92 Å). Figure 1e,f show the selected area electron diffraction patterns of Gd_2_InSbO_7_ and Gd_2_FeSbO_7_, respectively. It could be seen that Gd_2_InSbO_7_ and Gd_2_FeSbO_7_ crystallized with a cubic crystal system, and the lattice parameters *α* of Gd_2_InSbO_7_ and Gd_2_FeSbO_7_ were proved to be 10.449546 Å and 10.276026 Å, respectively. According to the calculation results from Figure 1e,f the main diffraction peaks (222), (440), (400) and (622) for Gd_2_InSbO_7_ and Gd_2_FeSbO_7_ could be found and indexed within Figure 1e,f.

Our X-ray diffraction outcomes showed that Gd_2_FeSbO_7_, Gd_2_InSbO_7_ and Bi_2_InTaO_7_ crystallized with the same pyrochlore-type structure. The cubic system structure with space group *Fd3m* for Bi_2_InTaO_7_ remained unchanged, with Ta^5+^ being substituted by Sb^5+^ and with Bi^3+^ being substituted by Gd^3+^. The cubic system structure with space group *Fd3m* for Bi_2_InTaO_7_ also remained unchanged with Ta^5+^ being substituted by Sb^5+,^ with In^3+^ being substituted by Fe^3+^ and with Bi^3+^ being substituted by Gd^3+^. The result of refinements for Gd_2_FeSbO_7_ generated the unweighted *R* factors, *R*_P_ = 16.20% with space group *Fd3m*. Similarly, the result of refinements for Gd_2_InSbO_7_ generated the unweighted *R* factors, *R*_P_ = 12.13% with space group *Fd3m*. Zou *et al.* [46] refined the crystal structure of Bi_2_InNbO_7_ and obtained a large *R* factor, which was owing to a slightly modified structure model for Bi_2_InNbO_7_. Based on the high purity of the precursors that were used in this study and the EDS results that did not trace any other elements, it was unlikely that the observed space groups originated from the presence of impurities. Therefore, it was suggested that the slightly high *R* factor for Gd_2_FeSbO_7_ or Gd_2_InSbO_7_ was due to a slightly modified structure model of Gd_2_FeSbO_7_ or Gd_2_InSbO_7_. It should be emphasized that the defects or the disorder/order of a fraction of the atoms could result in the change of structures, including different bond-distance distributions, thermal displacement parameters and/or occupation factors for some of the atoms.

The XPS spectra of Gd_2_FeSbO_7_ and Gd_2_InSbO_7_ were obtained. The various elemental peaks, which are corresponding to specific binding energies, are given in Table 3. The results further suggested that the oxidative valence state of Gd, Fe, Sb and O ions from Gd_2_FeSbO_7_ were +3, +3, +5 and −2, respectively. For Gd_2_FeSbO_7_, the mean atomic ratios of Gd: Fe: Sb: O were 2.00: 1.02: 0.99: 6.96 based on averaging our XPS, SEM-EDS and XFS results. Similarly, the oxidation state of Gd, In, Sb and O ions from Gd_2_InSbO_7_ were +3, +3, +5 and −2, respectively. For Gd_2_InSbO_7_, the mean atomic ratios of Gd: In: Sb: O were 2.00: 1.03: 0.97: 6.98 based on averaging our XPS, SEM-EDS and XFS results. Consequently, it could be deduced that the final materials were of high purity under our preparation conditions. It was noteworthy that neither shoulders nor widening of any XPS peaks of Gd_2_FeSbO_7_ or Gd_2_InSbO_7_ were observed, suggesting the absence of any other phases.

**Figure 4:**
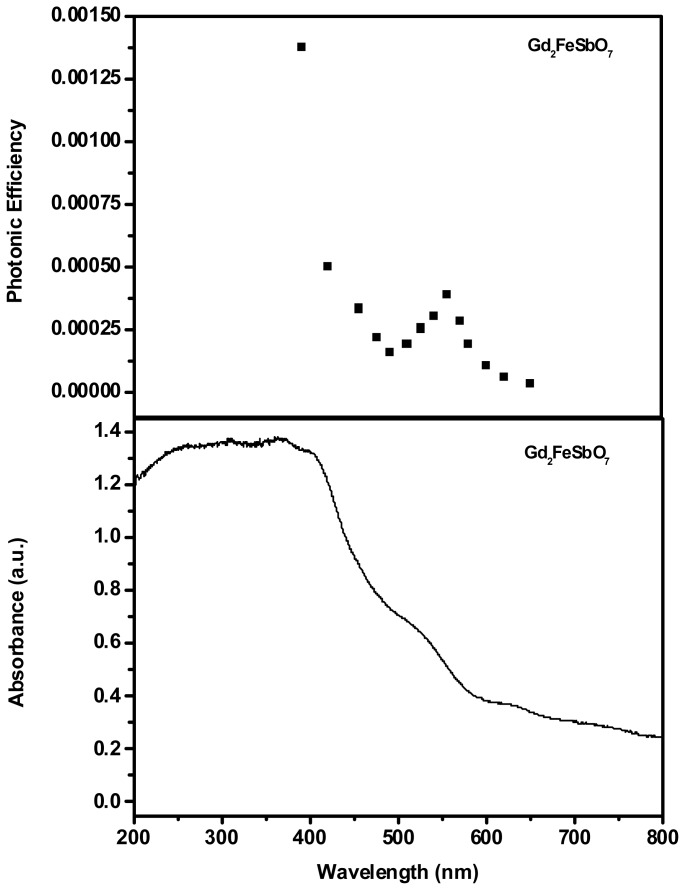
Upper trace: action spectra of rhodamine B degradation with Gd_2_FeSbO_7_ as catalyst under visible light irradiation. Lower trace: absorption spectra of Gd_2_FeSbO_7_.

**Figure 5:**
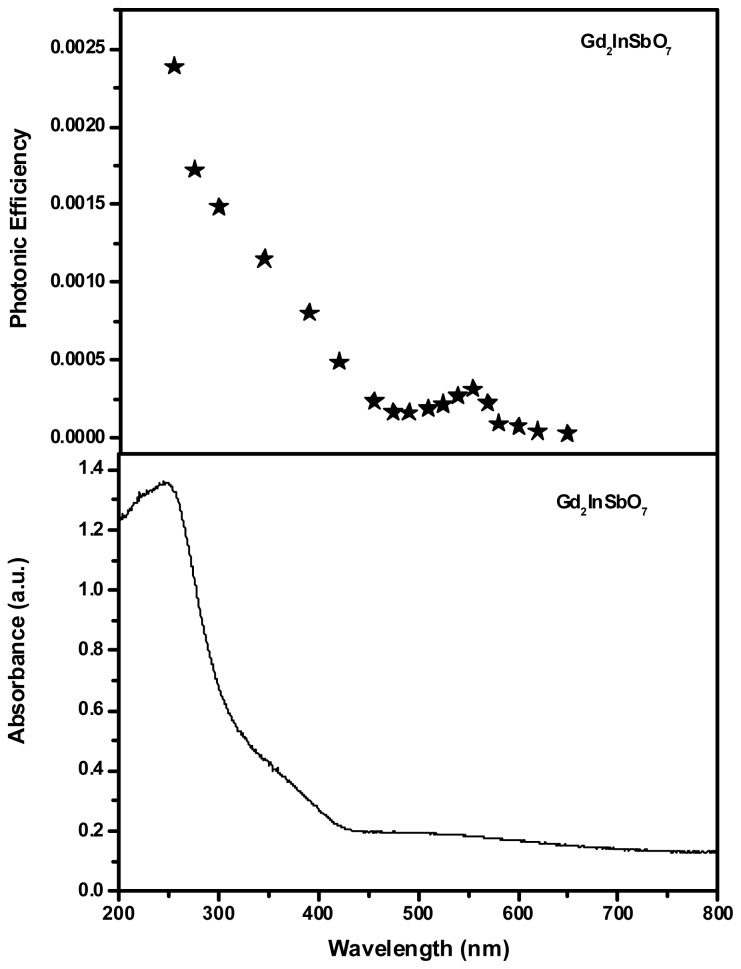
Upper trace: action spectra of rhodamine B degradation with Gd_2_InSbO_7_ as catalyst under visible light irradiation. Lower trace: absorption spectra of Gd_2_InSbO_7_.

Figures 4 and 5 present the absorption spectra of Gd_2_FeSbO_7_ and Gd_2_InSbO_7_, respectively. In contrast to the well-known TiO_2_, whose absorption edge was less than 380 nm, the absorption edges of newly synthesized Gd_2_FeSbO_7_ and Gd_2_InSbO_7_ were found to be 586 nm and 428 nm, respectively, which were in the visible region of the spectrum. It was noteworthy that the apparent absorption (defined hereby as 1-transmission) could not take into consideration reflection and scattering. As a result, the apparent absorbance at sub-bandgap wavelengths (600 to 800 nm for Gd_2_FeSbO_7_ and 425 to 800 nm for Gd_2_InSbO_7_) was higher than zero.

**Figure 6:**
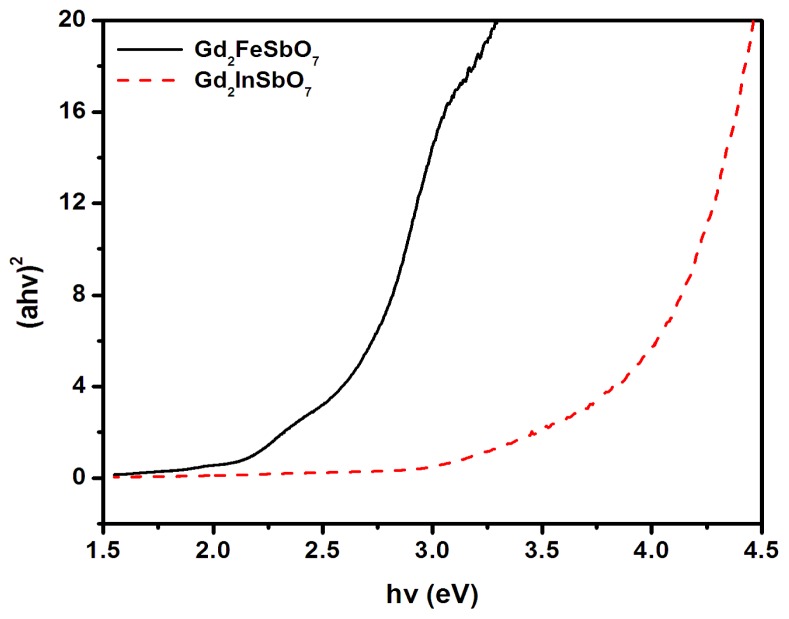
Plot of (*αhν*)^2^*versus hν* for Gd_2_FeSbO_7_ and Gd_2_InSbO_7_.

For a crystalline semiconductor compound, the optical absorption near the band edge followed the equation [47,48]: *αhν* = A (*hν*-*E*_g_)*^n^*. Here, A, *α*, *E*_g_ and *ν* denoted proportional constant, absorption coefficient, band gap and light frequency, respectively. In this equation, *n* determined the character of the transition in a semiconductor compound. *E*_g_ and *n* could be calculated by the following steps: (i) plotting ln(*αhν*) *versus* ln(*hν*-*E*_g_) assuming an approximate value of *E*_g_, (ii) deducing the value of *n* based on the slope in this graph and (iii) refining the value of *E*_g_ by plotting (*αhν*)*^1/n^versus hν* and extrapolating the plot to (*αhν*)*^1/n^* = 0. According to this method, Figure 6 shows the plot of (*αhν*)*^1/n^versus hν* for Gd_2_FeSbO_7_ and Gd_2_InSbO_7_. It was evidently to be found from Figure 6 that the values of *E*_g_ for Gd_2_FeSbO_7_ and Gd_2_InSbO_7_ were calculated to be 2.151 eV and 2.897 eV, respectively, while the values of *n* for Gd_2_FeSbO_7_ and Gd_2_InSbO_7_ were calculated to be 0.50 and 0.50, respectively, indicating that Gd_2_FeSbO_7_ possessed a narrower band gap compared with that of Gd_2_InSbO_7_.

### 2.2. Photocatalytic Activity

**Figure 7:**
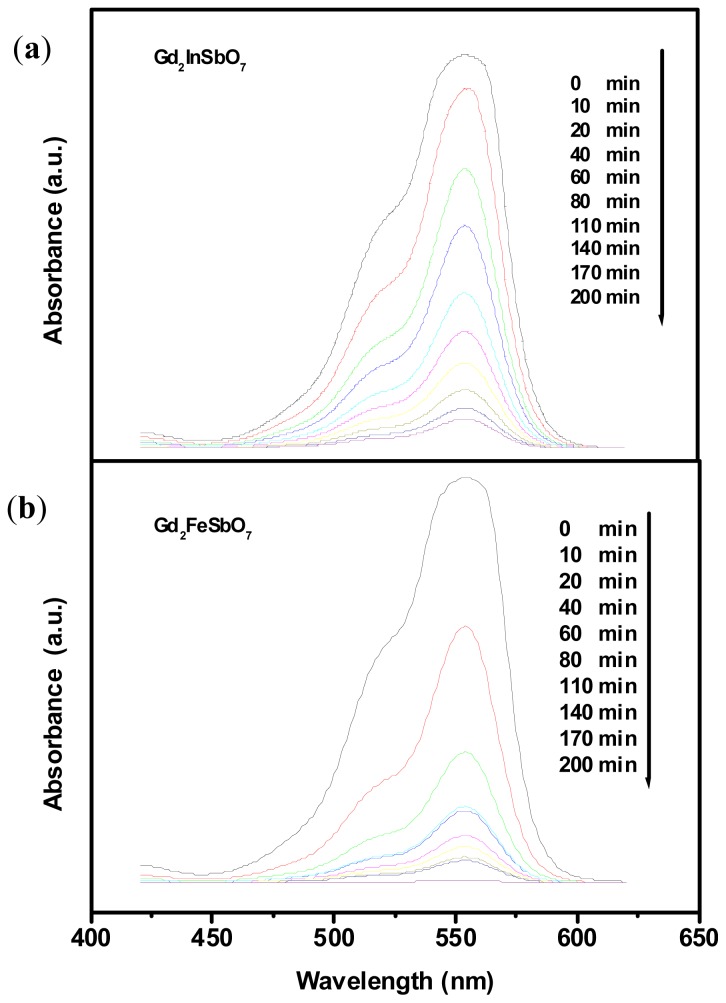
Temporal spectral changes of aqueous rhodamine B due to visible light irradiation in the presence of (**a**) Gd_2_InSbO_7_ or (**b**) Gd_2_FeSbO_7_.

Generally, the photocatalytic process by semiconductors begins with the direct absorption of supra-bandgap photons and the generation of electron–hole pairs within semiconductor particles. This is followed by diffusion of the charge carriers to the surface of the semiconductor particle. Changes in the UV-Vis spectrum of rhodamine B (RhB) upon exposure to visible light (*λ* > 400 nm) with the presence of Gd_2_FeSbO_7_ or Gd_2_InSbO_7_ are depicted in Figure 7a,b, respectively. The measurements were performed under oxygen-saturation conditions ([O_2_]_sat_ = 1.02 × 10^−3^ M). The degradation of RhB did not occur in darkness within the Gd_2_FeSbO_7_/RhB suspension or Gd_2_InSbO_7_/RhB suspension or Bi_2_InTaO_7_/RhB suspension or RhB suspension. It could be seen from Figure 7a and Figure 7b that a reduction of typical RhB peaks at 553.5 nm and 525 nm was clearly noticed. The results showed that the initial degradation rate of RhB was about 2.413 × 10^−9^ mol L^−1^ s^−1^, and the initial photonic efficiency was estimated to be 0.05069% (*λ* = 420 nm) for Gd_2_FeSbO_7_. Similarly, the initial degradation rate of RhB was about 2.322 × 10^−9^ mol L^−1^ s^−1^, and the initial photonic efficiency was estimated to be 0.04877% (*λ* = 420 nm) for Gd_2_InSbO_7_. For Bi_2_InTaO_7_, after visible light irradiation for 200 min, the RhB concentration decreased only from 0.0293 mM to 0.0161 mM, and the initial degradation rate of RhB was about 1.1 × 10^−9^ mol L^−1^ s^−1^. The initial photonic efficiency was estimated to be 0.02311% (*λ* = 420 nm) for Bi_2_InTaO_7_. By contrast, the photonic efficiency of Bi_2_InTaO_7_ was inferior to that of Gd_2_FeSbO_7_ or Gd_2_InSbO_7_.

**Figure 8:**
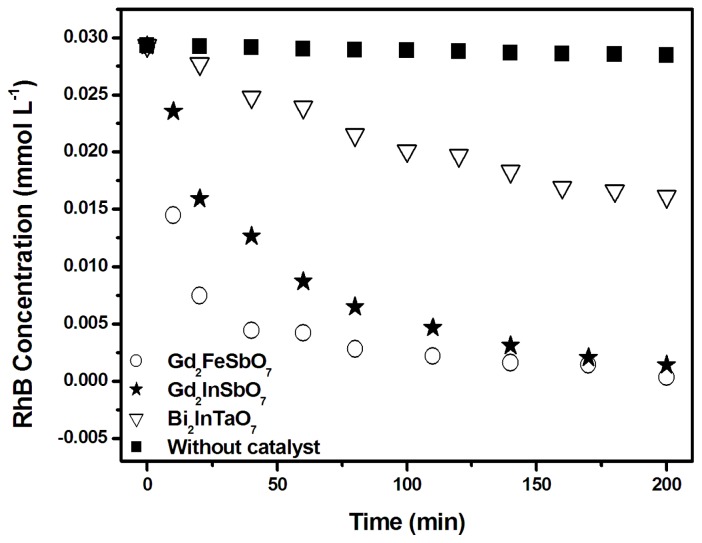
Photocatalytic degradation of rhodamine B under visible light irradiation in the presence of Gd_2_FeSbO_7_, Gd_2_InSbO_7_, Bi_2_InTaO_7_, as well as in the absence of a photocatalyst.

The kinetics of RhB degradation deduced according to the spectral changes under visible light irradiation are shown in Figure 8, which describes the kinetics not only with Gd_2_FeSbO_7_, Gd_2_InSbO_7_ and Bi_2_InTaO_7_ as catalysts, but also without any photocatalyst. As expected, reduction of RhB signal in the controlled measurements in the absence of a photocatalyst was promising. In addition, the photodegradation removal rate of RhB was 90.35%, 77.80% and 26.62% after visible light irradiation for 80 min with Gd_2_FeSbO_7_, Gd_2_InSbO_7_ and Bi_2_InTaO_7_ as catalysts, respectively. Complete removal of rhodamine B was observed after visible light irradiation for 225 min or 260 min with Gd_2_FeSbO_7_ or Gd_2_InSbO_7_ as photocatalyst. Based on the above outcomes, it was much faster for Gd_2_FeSbO_7_ and Gd_2_InSbO_7_ to photodegrade RhB compared with Bi_2_InTaO_7_, and the photocatalytic degradation activity of Gd_2_FeSbO_7_ or Gd_2_InSbO_7_ for degrading RhB was higher than that of Bi_2_InTaO_7_; moreover, Gd_2_FeSbO_7_ showed higher photocatalytic degradation activity than Gd_2_InSbO_7_. The main reason was that the lattice parameter α = 10.746410 Å for Bi_2_InTaO_7_ was larger than the lattice parameter α = 10.449546 Å for Gd_2_InSbO_7_, and the lattice parameter α = 10.449546 Å for Gd_2_InSbO_7_ was larger than the lattice parameter α = 10.276026 Å for Gd_2_FeSbO_7_, which probably resulted in a decrease for the migration distance of photogenerated electrons and holes to reach the reaction site on the photocatalyst surface; subsequently, the creation of more active sites was realized. As a result, it would probably improve the photocatalytic activities by decreasing the lattice parameter of the photocatalyst.

**Figure 9:**
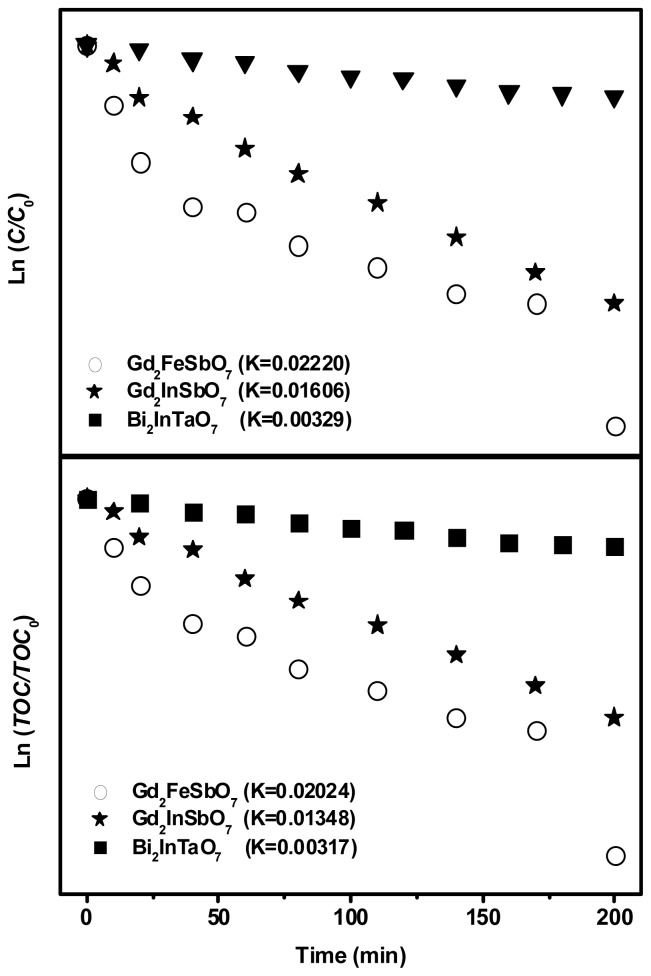
Observed first-order kinetic plots for the photocatalytic degradation of rhodamine B with Gd_2_FeSbO_7_, Gd_2_InSbO_7_ or Bi_2_InTaO_7_ as catalyst under visible light irradiation.

The first order nature of the photocatalytic degradation kinetics with Gd_2_FeSbO_7_, Gd_2_InSbO_7_ and Bi_2_InTaO_7_ as catalysts is clearly exhibited in Figure 9, which describes a linear correlation between ln(*C/C_0_*) (or ln(*TOC/TOC_0_*)) and the irradiation time for the photocatalytic degradation of RhB under visible light irradiation by using Gd_2_FeSbO_7_ or Gd_2_InSbO_7_ or Bi_2_InTaO_7_ as catalyst. Here, *C* represented the RhB concentration at time t, and *C_0_* represented the initial concentration of RhB. *TOC* represented the total organic carbon concentration at time t, and *TOC_0_* denoted the initial total organic carbon concentration. According to the relationship between ln(*C/C_0_*) and the irradiation time, the apparent first-order rate constant *k* was estimated to be 0.02220 min^−1^ with Gd_2_FeSbO_7_ as catalyst, 0.01606 min^−1^ with Gd_2_InSbO_7_ as catalyst and 0.00329 min^−1^ with Bi_2_InTaO_7_ as catalyst, indicating that Gd_2_FeSbO_7_ and Gd_2_InSbO_7_ were more effective than Bi_2_InTaO_7_ for the photocatalytic degradation of RhB under visible light irradiation. Meanwhile, Gd_2_FeSbO_7_ showed more effective photocatalytic activity for degrading RhB than Gd_2_InSbO_7_. According to the relationship between ln(*TOC/TOC_0_*) and the irradiation time, the apparent first-order rate constant *k* was estimated to be 0.02024 min^−1^ with Gd_2_FeSbO_7_ as catalyst, 0.01348 min^−1^ with Gd_2_InSbO_7_ as catalyst and 0.00317 min^−1^ with Bi_2_InTaO_7_ as catalyst, indicating that the photodegradation intermediate products of RhB were probably produced during the photocatalytic degradation of RhB under visible light irradiation.

**Figure 10:**
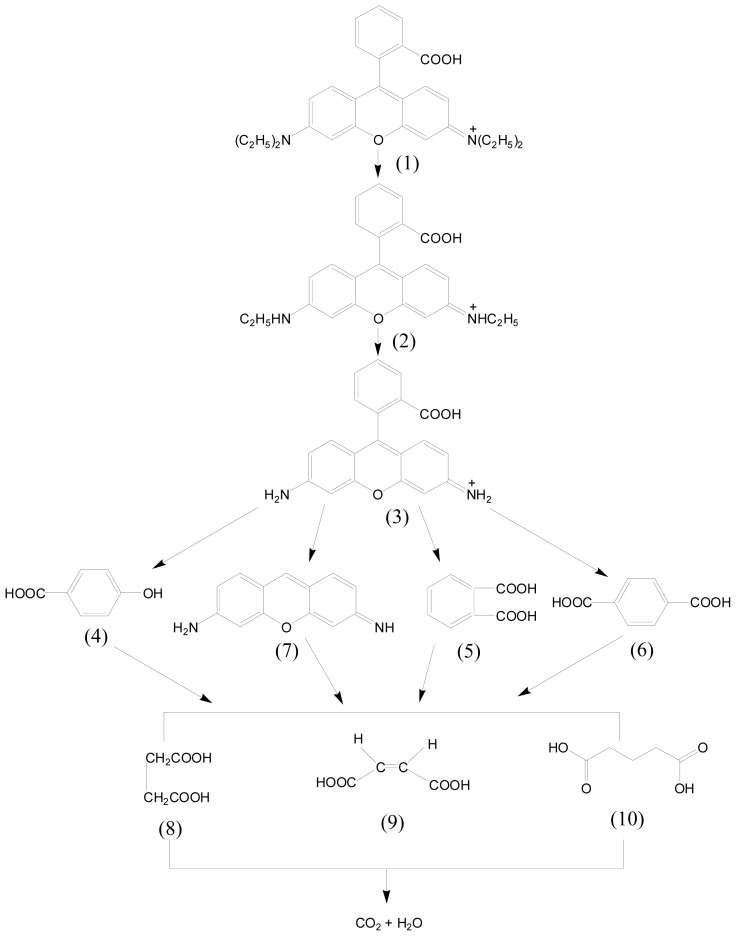
Suggested photocatalytic degradation pathway scheme for rhodamine B under visible light irradiation in the presence of Gd_2_FeSbO_7_ or Gd_2_InSbO_7_.

**Figure 11:**
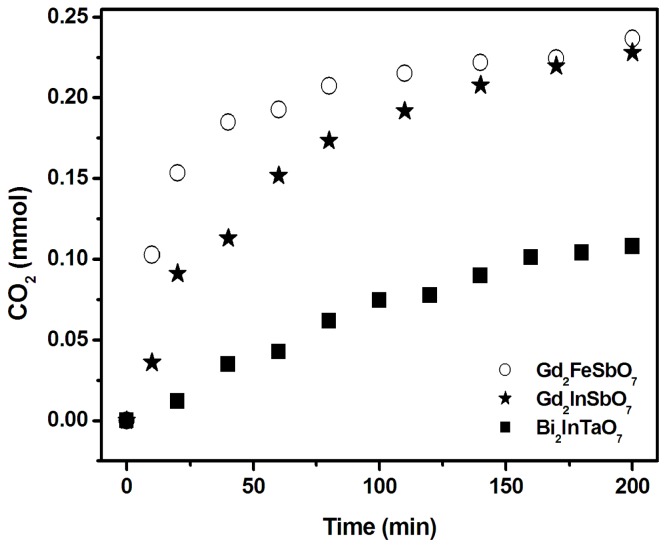
CO_2_ production kinetics during the photocatalytic degradation of rhodamine B with Gd_2_FeSbO_7_, Gd_2_InSbO_7_ or Bi_2_InTaO_7_ as catalyst under visible light irradiation.

The photodegradation intermediate products of RhB in our experiment were identified as succinic acid (*m/z* = 118), terephthalic acid (*m/z* = 166), pentanedioic acid, 3-Hydroxybenzoic acid (*m/z* = 138), 1,2-benzenedicarboxylic acid and maleic acid (*m/z* = 116). Based on the intermediate products detected in this work, a possible photocatalytic degradation pathway for RhB is proposed in Figure 10. This pathway was similar, but not identical, to the pathway proposed by Horikoshi *et al.* [49] for the photodegradation of RhB under ultraviolet light illumination assisted by microwave radiation with TiO_2_ as catalyst. According to the research from Zhang *et al.* [32], the photodegradation of RhB occurred through two competitive processes: one was *N*-demethylation and the other one was the destruction of the conjugated structure. Thus, we considered that chromophore cleavage, opening-ring and mineralization would be the main photocatalytic degradation pathway of RhB in our experiment. RhB was converted to smaller organic species, which were ultimately mineralized into inorganic products, such as CO_2_ and water. Figure 11 shows CO_2_ yields during the photocatalytic degradation of RhB with Gd_2_FeSbO_7_, Gd_2_InSbO_7_ or Bi_2_InTaO_7_ as catalyst under visible light irradiation. The results indicated that the yielded CO_2_ increased gradually with the increase of reaction time by using Gd_2_FeSbO_7_, Gd_2_InSbO_7_ or Bi_2_InTaO_7_ as catalyst. The production rate of CO_2_ with Gd_2_FeSbO_7_ or Gd_2_InSbO_7_ as catalyst was higher than that with Bi_2_InTaO_7_ as catalyst, which was in line with the absorption curves (Figures 4 and 5) of Gd_2_FeSbO_7_ and Gd_2_InSbO_7_. For example, after visible light irradiation for 200 min, the CO_2_ production was 0.2366 mmol with Gd_2_FeSbO_7_ as catalyst, 0.2280 mmol with Gd_2_InSbO_7_ as catalyst and 0.1080 mmol with Bi_2_InTaO_7_ as catalyst.

**Figure 12:**
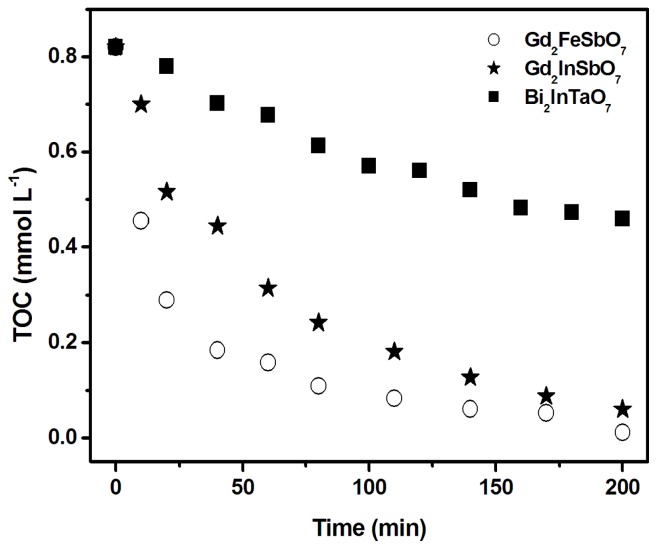
Disappearance of the total organic carbon (TOC) during the photocatalytic degradation of rhodamine B with Gd_2_FeSbO_7_, Gd_2_InSbO_7_ or Bi_2_InTaO_7_ as catalyst under visible light irradiation.

Figure 12 shows the change of the total organic carbon (TOC) for photocatalytic degradation of rhodamine B during visible light irradiation with Gd_2_FeSbO_7_, Gd_2_InSbO_7_ or Bi_2_InTaO_7_ as catalyst. The TOC measurements revealed the disappearance of organic carbon when the RhB solution containing Gd_2_FeSbO_7_, Gd_2_InSbO_7_ or Bi_2_InTaO_7_ was exposed under visible light irradiation. The results showed that 86.70%, 70.49% or 25.26% of TOC decrease was obtained after visible light irradiation for 80 min with Gd_2_FeSbO_7_, Gd_2_InSbO_7_ or Bi_2_InTaO_7_ as catalyst, respectively. The turnover numbers, which were the ratio between total amount of evolved gas and exhausted catalyst, were calculated to be more than 0.18 or 0.19 for Gd_2_FeSbO_7_ or Gd_2_InSbO_7_ after 200 min of reaction time under visible light irradiation. The reactions ceased when the light was turned off, indicating an obvious photic response.

The photocatalytic performance of Gd_2_FeSbO_7_ and Gd_2_InSbO_7_ was remarkable under visible light irradiation. This superior quality could be even more bracing if one considered the fact that the specific surface areas of Gd_2_FeSbO_7_ and Gd_2_InSbO_7_ were further smaller than that of titanium dioxide. In this experiment, BET isotherm measurements gave a specific surface area of 4.12 m^2^ g^−1^, 3.26 m^2^ g^−1^ and 1.26 m^2^ g^−1^ for Gd_2_FeSbO_7_, Gd_2_InSbO_7_ and Bi_2_InTaO_7_, respectively, which was almost 12-times smaller than that of TiO_2_ (46.24 m^2^ g^−1^). The particle sizes of Gd_2_InSbO_7_ and Gd_2_FeSbO_7_ were uneven. We provide SEM images with low magnification of Gd_2_InSbO_7_ and Gd_2_FeSbO_7_. Simultaneously, we give the TEM images of Gd_2_InSbO_7_ and Gd_2_FeSbO_7_, which represent the average particle size. Because the particle size of Gd_2_InSbO_7_ and Gd_2_FeSbO_7_ compounds were greater than 230nm, their specific surface areas were less than 5 m^2^ g^−1^.

There are three main reasons to choose Gd for producing Gd_2_FeSbO_7_ and Gd_2_InSbO_7_ in this paper. Firstly, the substitution of Bi^3+^ by Gd^3+^ and the substitution of Ta^5+^ by Sb^5+^ in Bi_2_InTaO_7_ will produce smaller catalyst particles and increase the specific surface area, because the radii of Gd^3+^, Bi^3+^, Ta^5+^ and Sb^5+^ are 1.053 Å, 1.17 Å, 0.74 Å and 0.60 Å, which shows that the radius of Gd^3+^ is smaller than the radius of Bi^3+^, and the radius of Sb^5+^ is smaller than the radius of Ta^5+^. Similarly, the substitution of Bi^3+^ by Gd^3+^, the substitution of In^3+^ by Fe^3+^ and the substitution of Ta^5+^ by Sb^5+^ in Bi_2_InTaO_7_ will produce smaller catalyst particles and increase the specific surface area, because the radii of In^3+^ and Fe^3+^ are 0.92 Å and 0.78 Å, which shows that the radius of Gd^3+^ is smaller than the radius of Bi^3+^, the radius of Fe^3+^ is smaller than the radius of In^3+^ and the radius of Sb^5+^ is smaller than the radius of Ta^5+^. Thus, the active sites and the catalytic activity will increase. Secondly, the smaller catalyst particles probably result in a decrease of the migration distance of photogenerated electrons and holes, which are formed under visible light irradiation from the grain interior to reach the reaction site on the photocatalyst surface. Subsequently, the photogenerated electrons and the photogenerated holes that reach the surface of the particles in unit time increase, and the creation of more active sites is realized. As a result, it will probably improve the photocatalytic activities with decreasing the particle size of the photocatalyst. Thirdly, some literatures reported that Gd-containing catalyst had excellent catalytic performance [50–53].

Figure 4 and Figure 5 show the action spectra of RhB degradation in the presence of Gd_2_FeSbO_7_ or Gd_2_InSbO_7_ under visible light irradiation. A clear photonic efficiency (0.03921% at its maximal point for Gd_2_FeSbO_7_ and 0.03145% at its maximal point for Gd_2_InSbO_7_) at wavelengths that corresponded to sub-Eg energies of the photocatalysts (*λ* from 425 to 800 nm) was observed for Gd_2_FeSbO_7_ and Gd_2_InSbO_7_. The existence of photonic efficiency at energies where photons were not absorbed by the photocatalysts, and the correlation between the low-energy action spectrum and the absorption spectrum of RhB clearly demonstrated that any photodegradation at wavelengths above 479 nm should be attributed to the photosensitization by RhB dye itself, the mechanism showing as follows:

(1) $${\text{RhB}}_{\left(\text{ads}\right)}\overset{\text{Visible light}}{\to }{{\text{RhB}}^{*}}_{\left(\text{ads}\right)}$$

(2) $${{\text{RhB}}^{*}}_{\left(\text{ads}\right)}+{\text{Gd}}_{2}{\text{FeSbO}}_{7}\;(\text{or }{\text{Gd}}_{2}{\text{InSbO}}_{7})\to {\text{Gd}}_{2}{\text{FeSbO}}_{7}\;(\text{or }{\text{Gd}}_{2}{\text{InSbO}}_{7})\;(\text{e})+{{\text{RhB}}^{+}}_{\left(\text{ads}\right)}$$

(3) $${\text{Gd}}_{2}{\text{FeSbO}}_{7}\;(\text{or }{\text{Gd}}_{2}{\text{InSbO}}_{7})\;(\text{e})+{\text{O}}_{2}\to {\text{Gd}}_{2}{\text{FeSbO}}_{7}\;(\text{or }{\text{Gd}}_{2}{\text{InSbO}}_{7})+\cdot {{\text{O}}_{2}}^{-}$$

According to this mechanism, RhB adsorbed on Gd_2_FeSbO_7_ or Gd_2_InSbO_7_ was excited by visible light irradiation. Subsequently an electron was injected from the excited RhB to the conduction band of Gd_2_FeSbO_7_ or Gd_2_InSbO_7_, where the electron was scavenged by molecular oxygen. Scheme I served to explain the results obtained with Gd_2_FeSbO_7_ or Gd_2_InSbO_7_ as catalyst under visible light irradiation, where Gd_2_FeSbO_7_ or Gd_2_InSbO_7_ might serve to reduce recombination of photogenerated electrons and holes by scavenging of electrons [54].

The situation was different below 479 nm, where the photonic efficiency correlated well with the absorption spectra of Gd_2_FeSbO_7_ and Gd_2_InSbO_7_. This result evidently showed that the mechanism, which was responsible for the photodegradation of RhB, went through band gap excitation of Gd_2_FeSbO_7_ or Gd_2_InSbO_7_. Although detailed experiments about the effect of oxygen and water on the degradation mechanism were not performed, it was sensible to assume that the mechanism in the first steps was similar to the observed mechanism for Gd_2_FeSbO_7_ or Gd_2_InSbO_7_ under supra-bandgap irradiation, namely showing below:

(4) $${\text{Gd}}_{2}{\text{FeSbO}}_{7}\;(\text{or }{\text{Gd}}_{2}{\text{InSbO}}_{7})\overset{\text{Visible light}}{\to }{\text{h}}^{+}+{\text{e}}^{-}$$

(5) $${\text{e}}^{-}+{\text{O}}_{2}\to \cdot {{\text{O}}_{2}}^{-}$$

(6) $${\text{h}}^{+}+{\text{OH}}^{-}\to \cdot \text{OH}$$

Previous luminescent studies had shown that the closer the M–O–M bond angle was to 180°, the more delocalized was the excited state [55]; As a result, the charge carriers could move easily in the matrix. The mobility of the photoinduced electrons and holes influenced the photocatalytic activity, because high diffusivity increased the probability that the photogenerated electrons and holes would reach the reactive sites of the catalyst surface. Based on the above results, the lattice parameter α = 10.276026 Å for Gd_2_FeSbO_7_ was smaller than the lattice parameter α = 10.449546 Å for Gd_2_InSbO_7_, thus the photoinduced electrons and holes inside Gd_2_FeSbO_7_ was easier and faster to reach the reactive sites of the Gd_2_FeSbO_7_ surface than those inside Gd_2_InSbO_7_; as a result, the photocatalytic degradation activity of Gd_2_FeSbO_7_ was higher than that of Gd_2_InSbO_7_. Moreover, in this experiment, for Gd_2_FeSbO_7_, the Fe–O–Fe bond angle was 116.424°; at the same time, for Gd_2_InSbO_7_, the In–O–In bond angle was 123.338°. The above results showed that the Fe–O–Fe bond angle of Gd_2_FeSbO_7_ or the In–O–In bond angle of Gd_2_InSbO_7_ was close to 180°, thus the photocatalytic activity of Gd_2_FeSbO_7_ or Gd_2_InSbO_7_ was accordingly higher. Moreover, the In–O–In bond angle of Gd_2_InSbO_7_ was larger than the Fe–O–Fe bond angle of Gd_2_FeSbO_7_, which led to an increase of photocatalytic activity for Gd_2_InSbO_7_ compared with that of Gd_2_FeSbO_7_. The crystal structures of Gd_2_FeSbO_7_, Gd_2_InSbO_7_ and Bi_2_InTaO_7_ were the same, but their electronic structures were considered to be a little different. For Gd_2_FeSbO_7_ or Gd_2_InSbO_7_, Sb or In was a 5*p*-block metal element, Gd was a 5*d*-block rare earth metal element and Fe was a 3*d*-block metal element, but for Bi_2_InTaO_7_, Ta was a 5*d*-block metal element and Bi was a 6*p*-block metal element, indicating that the photocatalytic activity might be affected not only by the crystal structure of the photocatalysts, but also by the electronic structure of the photocatalysts. According to the above analysis, the difference of RhB photocatalytic degradation among Gd_2_FeSbO_7_, Gd_2_InSbO_7_ and Bi_2_InTaO_7_ could be mainly attributed to the difference of their crystalline structure and electronic structure.

**Figure 13:**
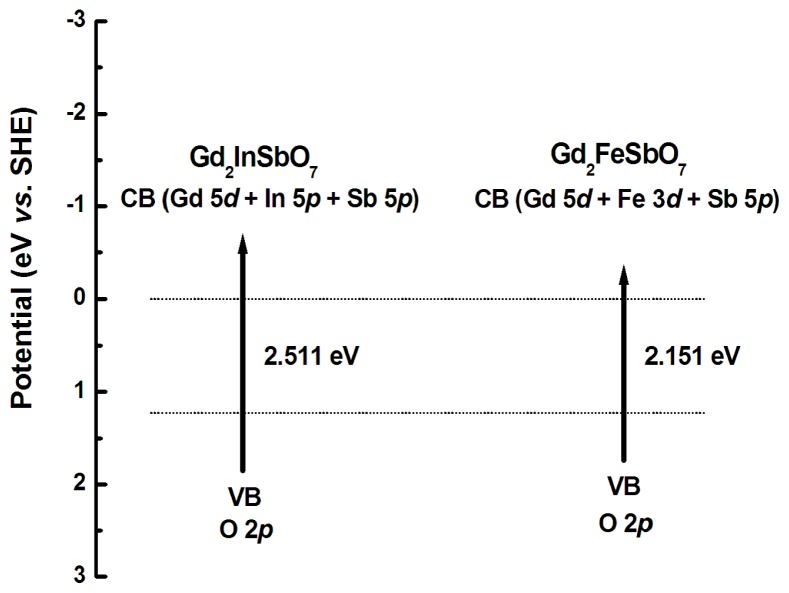
Suggested band structures of Gd_2_FeSbO_7_ and Gd_2_InSbO_7_.

Figure 13 shows the suggested band structures of Gd_2_FeSbO_7_ and Gd_2_InSbO_7_. The positions and width of the conduction band (CB) and the valence band (VB) were investigated by calculating the electronic band structure of Gd_2_FeSbO_7_ and Gd_2_InSbO_7_ with the plane-wave-based density functional method. The band structure calculations of Gd_2_FeSbO_7_ and Gd_2_InSbO_7_ were performed with the program of cambridge serial total energy package (CASTEP) and first-principles simulation. The CASTEP calculation was composed of the plane-wave pseudopotential total energy method according to the density functional theory. The generalized gradient approximation (GGA-PBE) and the geometry optimization were adopted. The selected unit cell for calculation was [Gd_2_FeSbO_7_]_2_ or [Gd_2_InSbO_7_]_2_. The pseudo-atomic calculations were accomplished for Gd_2_FeSbO_7_ with 5d^1^6s^2^ (Gd), 3d^6^4s^2^ (Fe), 5s^2^5p^3^ (Sb) and 2s^2^2p^4^ (O). The pseudo-atomic calculations were accomplished for Gd_2_InSbO_7_ with 5d^1^6s^2^ (Gd), 5s^2^5p^1^ (In), 5s^2^5p^3^ (Sb) and 2s^2^2p^4^ (O). The self-consistent field tolerance was 1.0 × 10^−6^ eV/atom. The core electrons were replaced by the ultra-soft pseudopotentials. The FFT grid of the basis was 40 × 30 × 30. The kinetic energy cutoff was 400 eV. 4 × 5 × 5 k-point, and the ultrasoft pseudopotential was applied in the above calculation. The total and partial density of the states of Gd_2_FeSbO_7_ and Gd_2_InSbO_7_ was taken into consideration. We determined the band structures of Gd_2_InSbO_7_ and Gd_2_FeSbO_7_ by borrowing ideas from the relevant literature [56–62]. Recently, the electronic structures of InMO_4_ (M = V, Nb and Ta) and BiVO_4_ were reported by Oshikiri *et al.* based on the first principles calculations [63]. The conduction bands of InMO_4_ (M = V, Nb and Ta) were mainly composed of a dominant *d* orbital component from V 3*d*, Nb 4*d* and Ta 5*d* orbitals, respectively. The valence bands of BiVO_4_ were composed of a small Bi 6*s* orbital component and a dominant O 2*p* orbital component. The band structures of Gd_2_FeSbO_7_ and Gd_2_InSbO_7_ should be similar to that of the above compounds. Therefore, we concluded that the conduction band of Gd_2_FeSbO_7_ was composed of Gd 5*d*, Fe 3*d* and Sb 5*p* orbital components; meanwhile, the valence band of Gd_2_FeSbO_7_ was composed of a small dominant O 2*p* orbital component. Similarly, the conduction band of Gd_2_InSbO_7_ was composed of Gd 5*d*, In 5*p* and Sb 5*p* orbital components; moreover, the valence band of Gd_2_InSbO_7_ was composed of a small dominant O 2*p* orbital component. Direct absorption of photons by Gd_2_FeSbO_7_ or Gd_2_InSbO_7_ could produce electron–hole pairs within the catalyst, indicating that larger energy than the band gap of Gd_2_FeSbO_7_ or Gd_2_InSbO_7_ was necessary for decomposing RhB by photocatalysis.

The presented results indicated that the Gd_2_FeSbO_7_ (or Gd_2_InSbO_7_)-visible light photocatalysis system might be regarded as a method for practical treatment of diluted colored waste water. Our Gd_2_FeSbO_7_ (or Gd_2_InSbO_7_)-visible light photocatalysis system could be utilized for decolorization, purification and detoxification in the textile, printing and dyeing industries within semi-arid countries. We designed Gd_2_FeSbO_7_ (or Gd_2_InSbO_7_)-visible light photocatalysis system without demanding chemical reagents or using the high pressure of oxygen or heating. The decolorized and detoxified water was submitted to our new system for treatment, and the results showed that the Gd_2_FeSbO_7_ (or Gd_2_InSbO_7_)-visible light photocatalysis system might provide a valuable treatment for purifying and reusing colored aqueous effluents.

## 3. Experimental Section

The novel photocatalysts were synthesized by a solid-state reaction method. Gd_2_O_3_, Bi_2_O_3_, Fe_2_O_3_, In_2_O_3_, Sb_2_O_5_ and Ta_2_O_5_ with purity of 99.99% (Sinopharm Group Chemical Reagent Co., Ltd., Shanghai, China) were used as starting materials. All powders were dried at 200 °C for 4 h before synthesis. For the sake of synthesizing Gd_2_FeSbO_7_, the precursors were stoichiometrically churned up, subsequently pressed into small columns and put into an alumina crucible (Shenyang Crucible Co., LTD, Shenyang, China). Eventually, calcination was performed at 1250 °C for 65 h in an electric furnace (KSL 1700X, Hefei Kejing Materials Technology CO., LTD, Hefei, China). Similarly, Gd_2_InSbO_7_ was prepared by calcination at 1320 °C for 65 h, and Bi_2_InTaO_7_ was prepared by calcination at 1050 °C for 46 h. The crystal structures of Gd_2_FeSbO_7_ and Gd_2_InSbO_7_ were analyzed by the powder X-ray diffraction method (D/MAX-RB, Rigaku Corporation, Tokyo, Japan) with Cu*K*α radiation (λ = 1.54056 angstrom). The data were collected at 295 K with a step-scan procedure in the range of 2θ = 10–100°. The step interval was 0.02°, and the time per step was 1.2 s. The chemical composition of Gd_2_InSbO_7_ and Gd_2_FeSbO_7_ was inspected by scanning electron microscope-X-ray energy dispersion spectrum (SEM-EDS, LEO 1530VP, LEO Electron Microscopy Inc., New York, NY, USA) and X-ray fluorescence spectrometer (XFS, ARL-9800, Thermo ARL, ARL Applied Research Laboratories S.A., Ecublens, Switzerland). The Gd^3+^ content, Sb^5+^ content, Fe^3+^ content, In^3+^ content and O^2−^ content of Gd_2_FeSbO_7_ and Gd_2_InSbO_7_ were detected by X-ray photoelectron spectroscopy (XPS, ESCALABMK-2, VG Scientific Ltd., East Grinstead, UK). The chemical composition within the depth profile of Gd_2_FeSbO_7_ or Gd_2_InSbO_7_ was examined by the argon ion denudation method when X-ray photoelectron spectroscopy was used. The optical absorption of Gd_2_FeSbO_7_ and Gd_2_InSbO_7_ was analyzed with an UV-visible spectrophotometer (Lambda 40, Perkin-Elmer Corporation, Waltham, MA, USA). The surface areas of Gd_2_InSbO_7,_ Gd_2_FeSbO_7_ and Bi_2_InTaO_7_ were surveyed by the Brunauer-Emmett-Teller (BET) method (MS-21, Quantachrome Instruments Corporation, Boynton Beach, FL, USA), with N_2_ adsorption at liquid nitrogen temperature. The particle sizes of the photocatalysts were measured by Malvern’s mastersize-2000 particle size analyzer (Malvern Instruments Ltd, Worcestershire, United Kingdom). The particle morphology of Gd_2_FeSbO_7_ and Gd_2_InSbO_7_ were measured by transmission electron microscope (TEM, Tecnal F20 S-Twin, FEI Corporation, Hillsboro, OR, USA).

The photocatalytic degradation of rhodamine B (RhB) (Tianjin Kermel Chemical Reagent Co., Ltd., Tianjin, China) was performed with Gd_2_InSbO_7_ or Gd_2_FeSbO_7_ or Bi_2_InTaO_7_ powder (0.8 g) suspended in RhB (300 mL 0.0293 mmol) solution by a pyrex glass cell (Jiangsu Yancheng Huaou Industry, Yancheng, China). Before visible light irradiation, the suspensions were magnetically stirred in darkness for 45 min to make sure the establishment of an adsorption/desorption equilibrium among Gd_2_FeSbO_7_, Gd_2_InSbO_7_, Bi_2_InTaO_7_, the RhB dye and atmospheric oxygen. The photocatalytic reaction system was composed of a 300 W Xe arc lamp, which was a light resource, with the main emission wavelength at 436 nm (Nanjing JYZCPST CO., LTD, Nanjing, China), a magnetic stirrer and a cut-off filter (λ > 400 nm, Jiangsu Nantong JSOL Corporation, Nantong, China). The Xe arc lamp was surrounded by a quartz jacket and was placed inside a photoreactor quartz vessel (5.8 cm in diameter and 68 cm in length) by which a suspension of RhB and the photocatalyst was circulated. An outer recycling water glass jacket maintained a near constant reaction temperature (22 °C), and the solution was stirred and aerated continuously. Two milliliters aliquots were sampled at various time intervals. The incident photon flux *I_o_* measured by a radiometer (Model FZ-A, Photoelectric Instrument Factory Beijing Normal University, Beijing, China) was determined to be 4.76 × 10^−6^ Einstein L^−1^ s^−1^ under visible light irradiation (wavelength range of 400–700 nm). The incident photon flux on the photoreactor was varied by adjusting the distance between the photoreactor and the Xe arc lamp. The initial pH value of the liquid was 7.0 and was not adjusted subsequently. The concentration of RhB was determined based on the absorption at 553.5 nm measured by an UV-Vis spectrophotometer (Lambda 40, Perkin-Elmer Corporation, Waltham, MA, USA). The inorganic products obtained from RhB degradation were analyzed by ion chromatograph (DX-300, Dionex Corporation, Sunnyvale, CA, USA). The identification of RhB and the degradation intermediate products of RhB were performed by gas chromatograph—mass spectrometer (GC-MS, HP 6890 series gas chromatograph, AT™ column, 20.3 m × 0.32 mm, ID of 0.25 μm) operating at 320 °C, which was connected to a HP 5973 mass selective detector and a flame ionization detector, with H_2_ as the carried gas. The intermediate products of RhB were measured by liquid chromatograph—mass spectrometer (LC-MS, Thermo Quest LCQ Duo, Atlanta, GA, USA, Beta Basic-C_18_ HPLC column: 150 × 2.1 mm, ID of 5 μm, Finnigan, Thermo, Atlanta, GA, USA). Here, post-photocatalysis solution (20 μL) was injected automatically into the LC-MS system. The eluent contained 60% methanol and 40% water, and the flow rate was 0.2 mL min^−1^. MS conditions included an electrospray ionization interface, a capillary temperature of 27 °C with a voltage of 19.00 V, a spray voltage of 5000 V and a constant sheath gas flow rate. The spectrum was acquired in the negative ion scan mode, sweeping the *m/z* range from 50 to 600. Evolution of CO_2_ was analyzed with an intersmat™ IGC120-MB gas chromatograph equipped with a porapack Q column (3 m in length and an inner diameter of 0.25 in.), which was connected to a catharometer detector.

The total organic carbon (TOC) concentration was determined with a TOC analyzer (TOC-5000, Shimadzu Corporation, Kyoto, Japan). The photonic efficiency was calculated according to the following equation [64]:

(7) $$\xi =R/{\text{I}}_{0}$$

where ϕ was the photonic efficiency (%), *R* was the rate of RhB degradation (Mol L^−1^ s^−1^) and *I_o_* was the incident photon flux (Einstein L^−1^ s^−1^).

## 4. Conclusions

Gd_2_FeSbO_7_ and Gd_2_InSbO_7_ were prepared by the solid-state reaction method for the first time. The structural, optical absorption and photocatalytic properties of Gd_2_FeSbO_7_ and Gd_2_InSbO_7_ were investigated and compared with Bi_2_InTaO_7_. XRD results indicated that Gd_2_FeSbO_7_ and Gd_2_InSbO_7_ crystallized with the pyrochlore-type structure, cubic crystal system and space group *Fd3m*. The lattice parameters of Gd_2_FeSbO_7_ and Gd_2_InSbO_7_ were found to be α = 10.276026 Å and α = 10.449546 Å. The band gaps of Gd_2_FeSbO_7_ and Gd_2_InSbO_7_ were estimated to be about 2.151 eV and 2.897 eV, indicating that Gd_2_FeSbO_7_ and Gd_2_InSbO_7_ showed a strong optical absorption in the visible light region (λ > 400 nm). Photocatalytic degradation of aqueous RhB was observed under visible light irradiation in the presence of Gd_2_FeSbO_7_ and Gd_2_InSbO_7_ accompanied with the formation of final products, such as CO_2_ and water. Complete removal of carbon from RhB was obtained, as indicated from TOC and CO_2_ yield measurements, with Gd_2_FeSbO_7_ or Gd_2_InSbO_7_ as catalyst under visible light irradiation. A Gd_2_FeSbO_7_ or (Gd_2_InSbO_7_) visible light photocatalysis system could be regarded as an effective way for treating textile industry wastewater. Gd_2_FeSbO_7_ or Gd_2_InSbO_7_ also showed higher photocatalytic activity compared with Bi_2_InTaO_7_ for RhB photocatalytic degradation under visible light irradiation. Moreover, Gd_2_FeSbO_7_ exhibited higher catalytic activity than Gd_2_InSbO_7_. The photocatalytic degradation of RhB was in line with the first-order reaction kinetics. The apparent first-order rate constant *k* was 0.01606, 0.02220 and 0.00318 min^−1^ with Gd_2_FeSbO_7_, Gd_2_InSbO_7_ or Bi_2_InTaO_7_ as catalyst. The possible photocatalytic degradation pathway of RhB was proposed under visible light irradiation.

## Figures and Tables

**Table 1:** Structural parameters of Gd_2_FeSbO_7_ prepared by the solid state reaction method.

Atom	*x*	*y*	*z*	Occupation factor
Gd	0.00000	0.00000	0.00000	1.0
Fe	0.50000	0.50000	0.50000	0.5
Sb	0.50000	0.50000	0.50000	0.5
O(1)	−0.20249	0.12500	0.12500	1.0
O(2)	0.12500	0.12500	0.12500	1.0

**Table 2:** Structural parameters of Gd_2_InSbO_7_ prepared by the solid state reaction method.

Atom	*x*	*y*	*z*	Occupation factor
Gd	0.00000	0.00000	0.00000	1.0
In	0.50000	0.50000	0.50000	0.5
Sb	0.50000	0.50000	0.50000	0.5
O(1)	−0.15469	0.12500	0.12500	1.0
O(2)	0.12500	0.12500	0.12500	1.0

**Table 3:** Binding energies (BE) for key elements.

In_3d5/2_ BE (eV)	Sb_3d5/2_ BE (eV)	Fe_2p3/2_ BE (eV)	Gd_4d5/2_ BE (eV)	O_1s_ BE (eV)
	530.85	710.81	143.93	530.35
444.65	530.82		143.85	530.12
